# Supplementary figures and images for: Intratumor heterogeneity defines treatment‐resistant HER2+ breast tumors
Source: Mol Oncol. 2018 Sep 21;12(11):1838–55. doi: 10.1002/1878-0261.12375 (PMC6210052; doi:10.1002/1878-0261.12375)

Supplementary Figure 1: Images from all pre-treatment biopsies

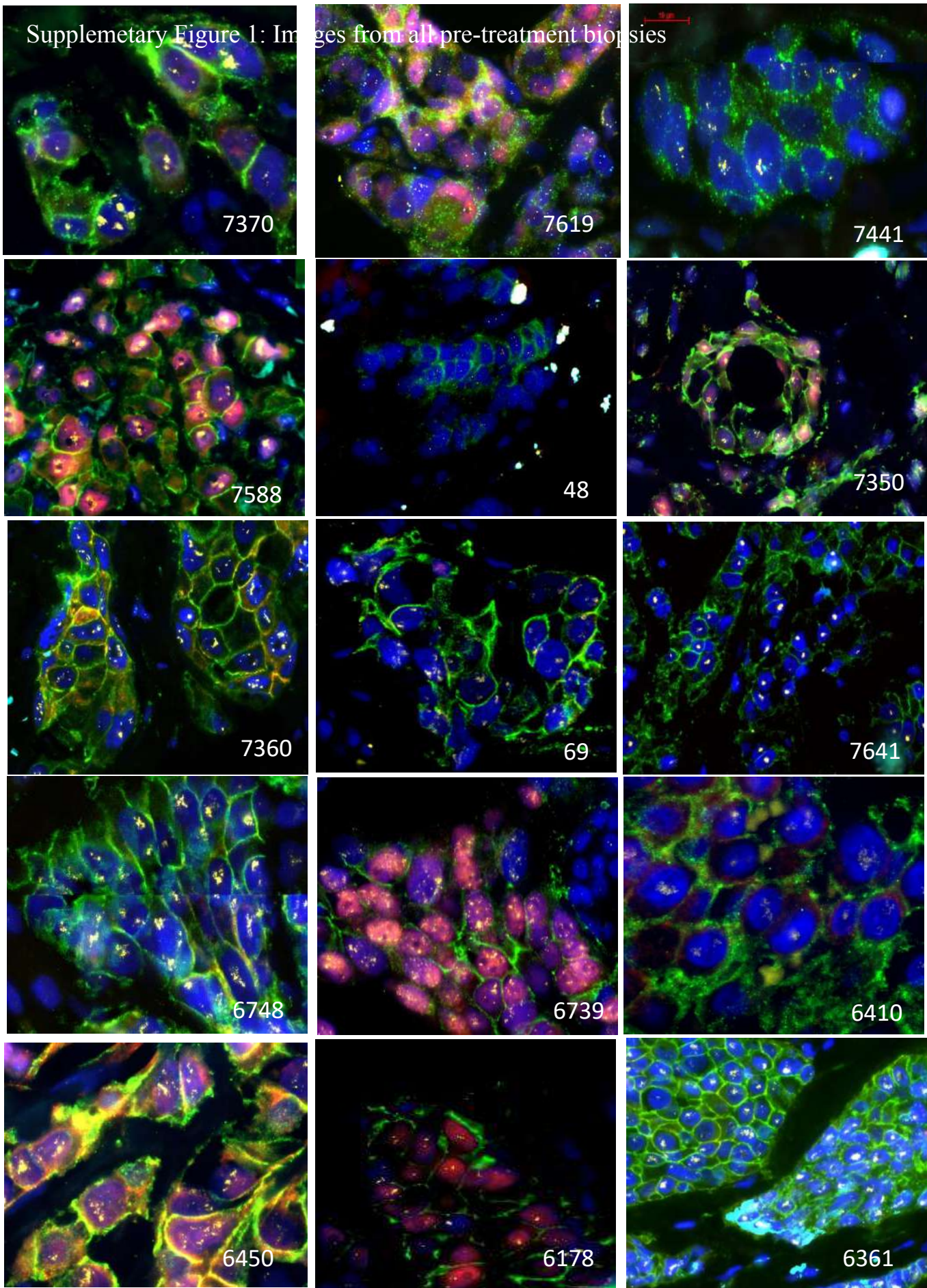

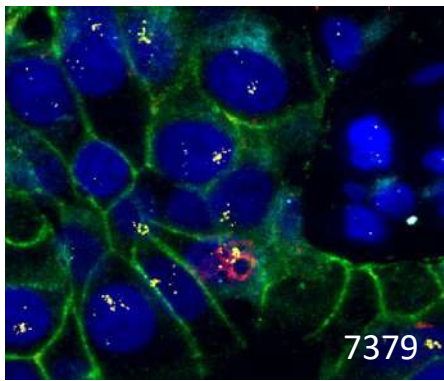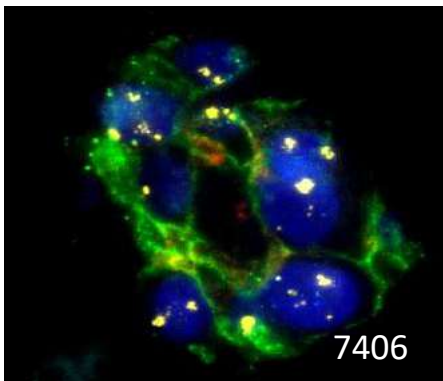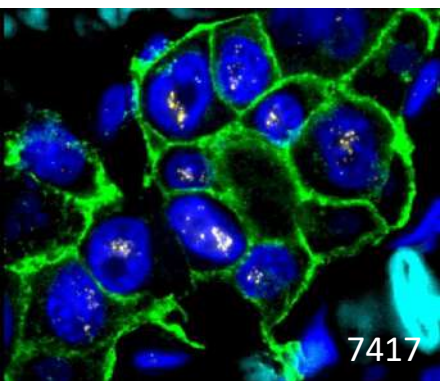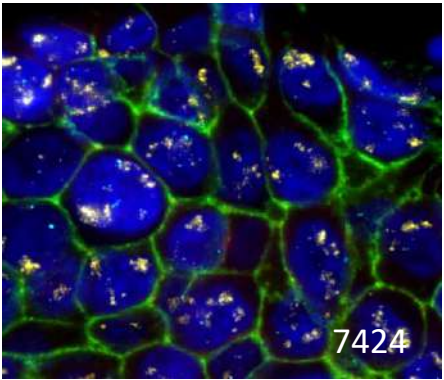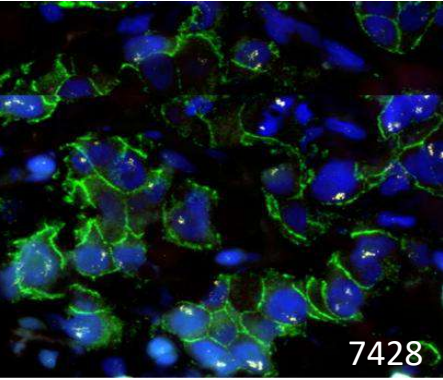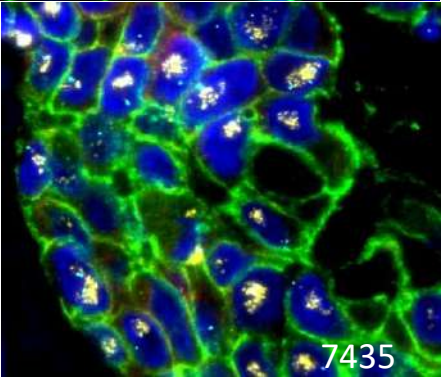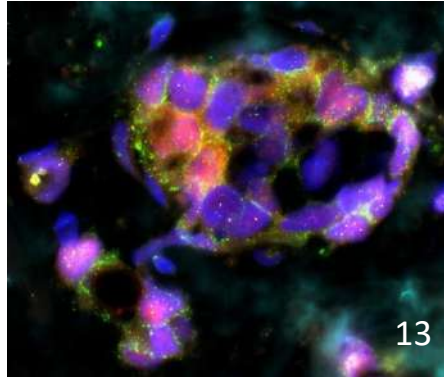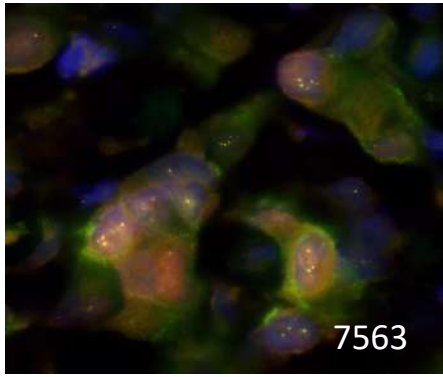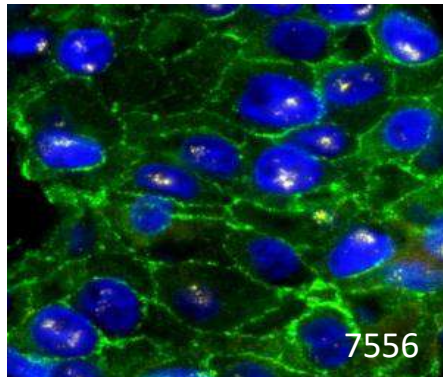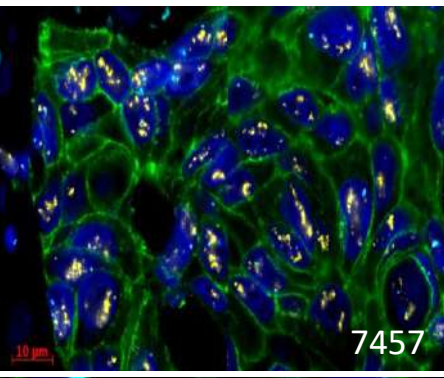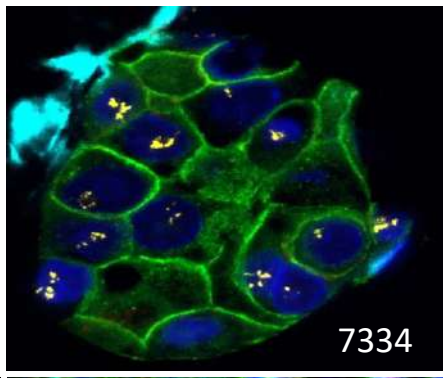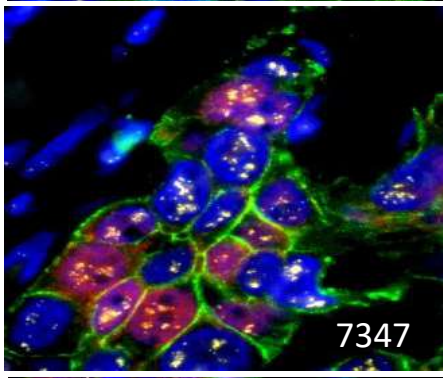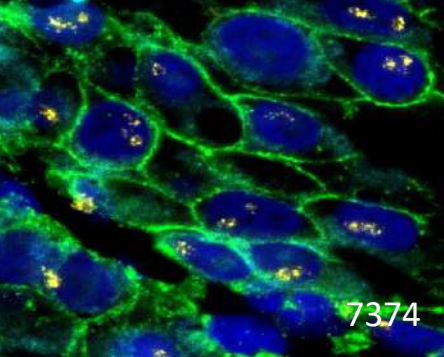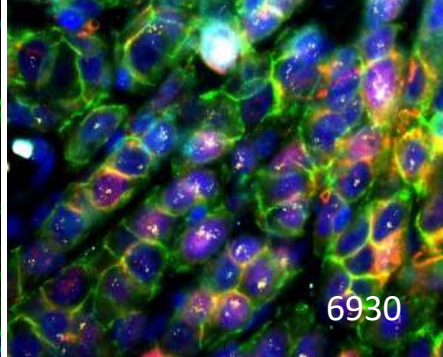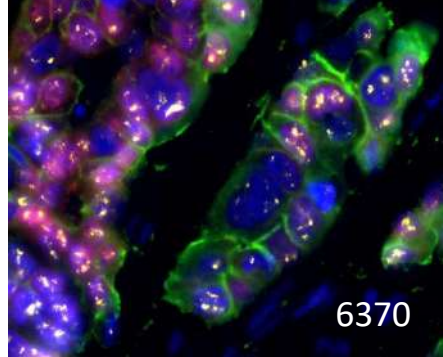

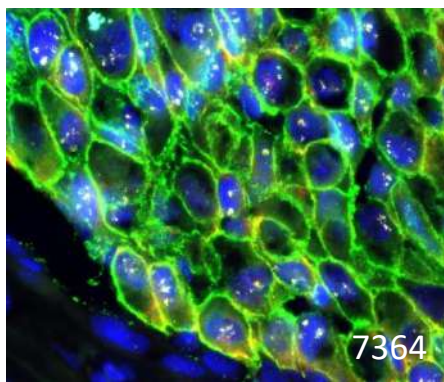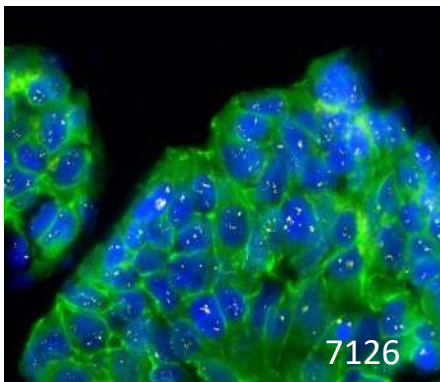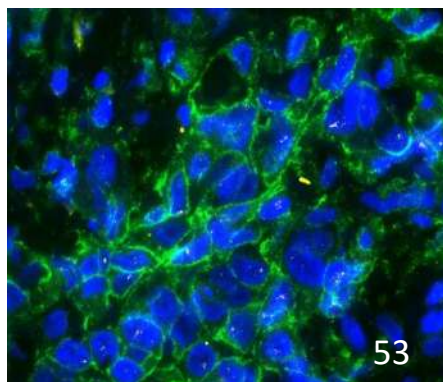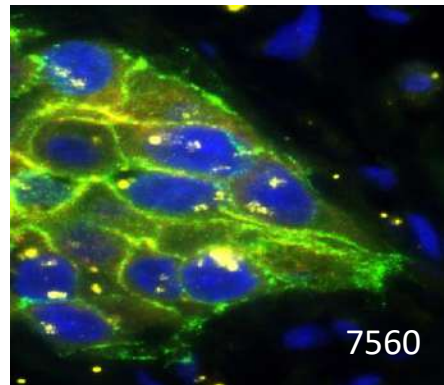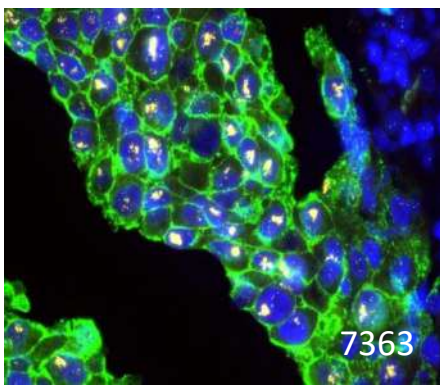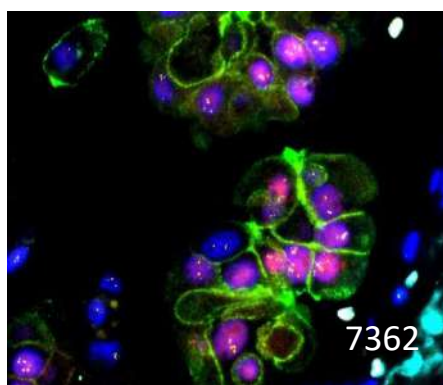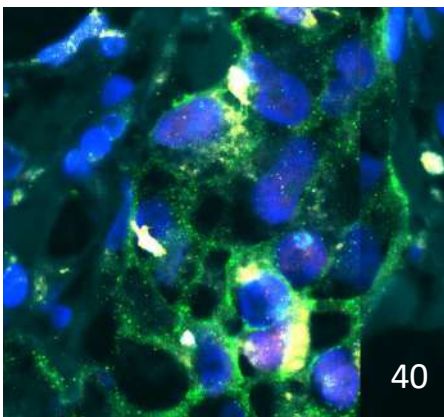

Supplement: Supplementary file 1 — Fig. S1. Images from all pre‐treatment biopsies. [file MOL2-12-1838-s001.pdf]
