# Supplementary material for: Intratumor heterogeneity defines treatment‐resistant HER2+ breast tumors
Source: Mol Oncol. 2018 Sep 21;12(11):1838–55. doi: 10.1002/1878-0261.12375 (PMC6210052; doi:10.1002/1878-0261.12375)

Supplementary Figure 2

A) Analyses of the pre-treatment samples (n=37) stratified by treatment response (pCR, n=12 and non-pCR, n=25).

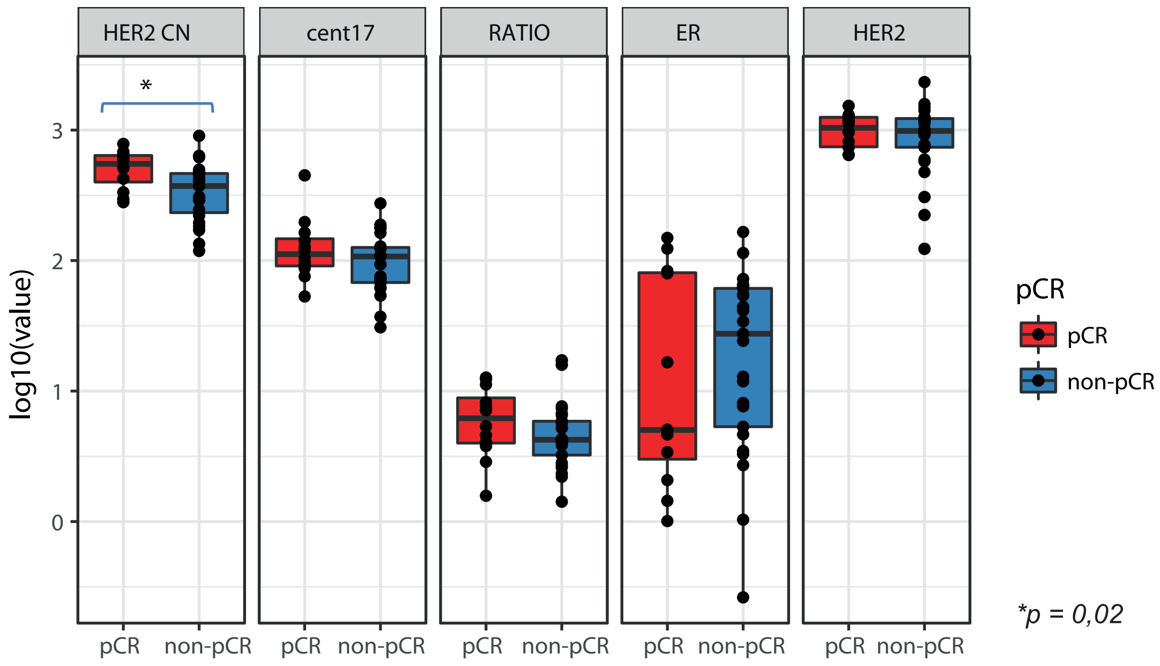

B) Risk of relapse and breast cancer related death with regard to the level of ER+ cells in pre-treatment biopsies.

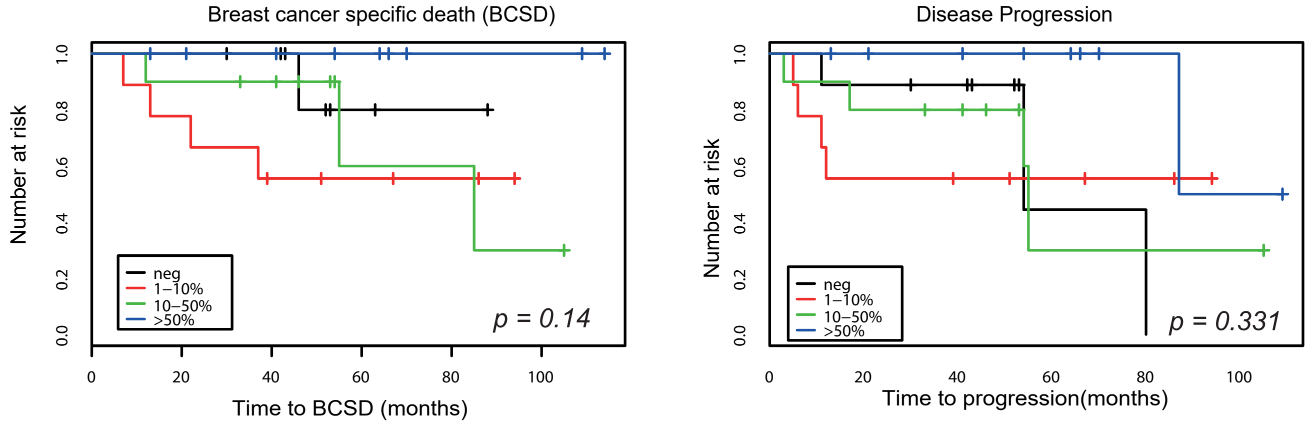

Supplement: Supplementary file 2 — Fig. S2. (A) Analyses of the pre‐treatment samples (n = 37) stratified by treatment response (pCR, n = 12 and non‐pCR, n = 25), (B) Risk of relapse and breast cancer related death with regard to the level of ER+ cells in pre‐treatment biopsies. [file MOL2-12-1838-s002.pdf]
