# Supplementary material for: Intratumor heterogeneity defines treatment‐resistant HER2+ breast tumors
Source: Mol Oncol. 2018 Sep 21;12(11):1838–55. doi: 10.1002/1878-0261.12375 (PMC6210052; doi:10.1002/1878-0261.12375)

Supplemental Figure 3
Distribution of ER and HER2 expression in each cell from all tumors separately (at two time points for non-pCR samples).
Dots representing cells from the pre-treatment samples are colored blue, from the post-treatment samples are colored red.
The x-axis represent ER receptor expression (intensity) and the y-axis represent the HER2 protein expression (intensity).

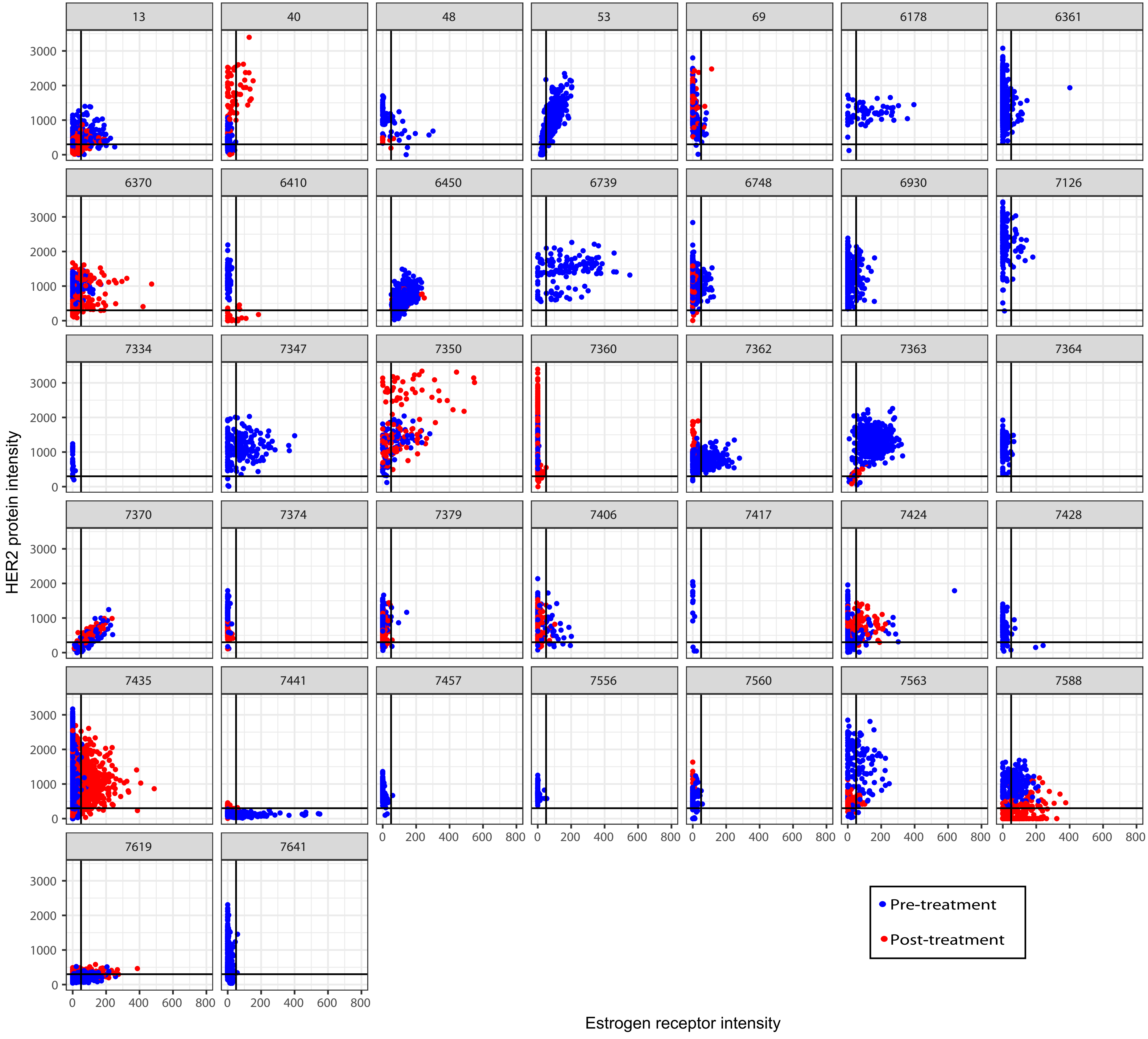

Supplement: Supplementary file 3 — Fig. S3. Distribution of ER and HER2 expression in each cell from all tumors separately (at two time‐points for non‐pCR samples). [file MOL2-12-1838-s003.pdf]
