# Supplementary material for: Intratumor heterogeneity defines treatment‐resistant HER2+ breast tumors
Source: Mol Oncol. 2018 Sep 21;12(11):1838–55. doi: 10.1002/1878-0261.12375 (PMC6210052; doi:10.1002/1878-0261.12375)

Supplementary Figure 5

A) Cluster analyses of 12 compined phenotype/*HER2* CN cell types, the three major groups identified are called PG1, PG2 and PG3.

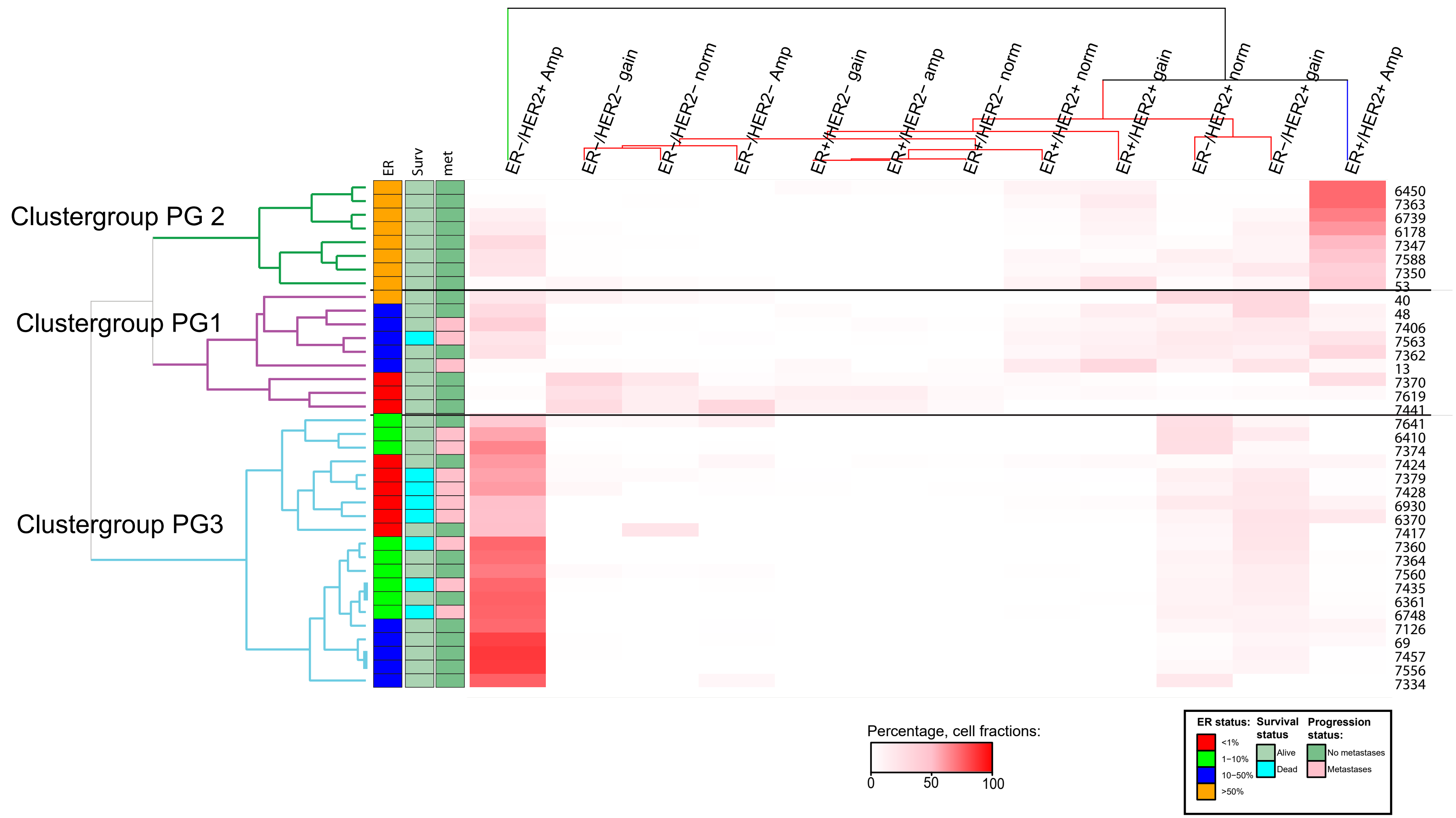

B) Risk for breast cancer specific death for patients in the tree clustergroups

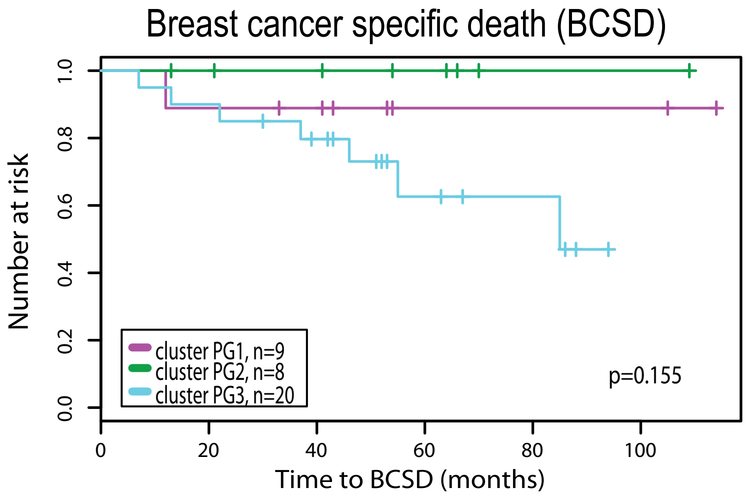

C) Risk for disease progression for patients in the tree clustergroups

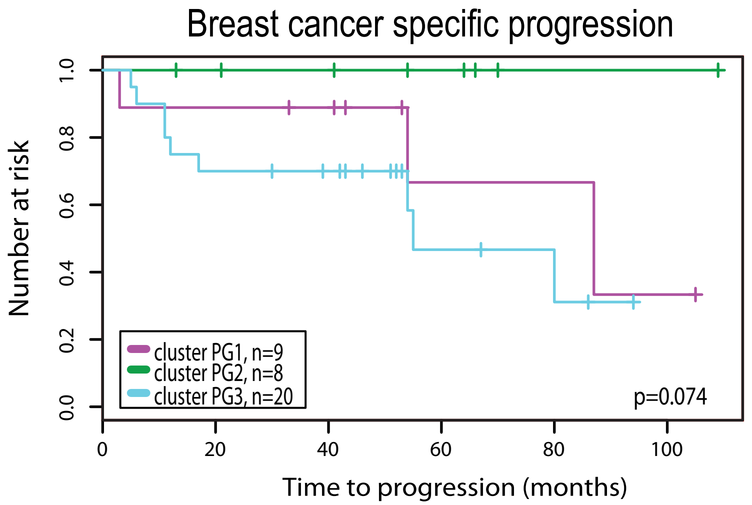

Supplement: Supplementary file 5 — Fig. S5. (A) Cluster analyses of the 12 combined phenotype/HER2 CN cell types on pre‐treatment samples (B) Risk of breast cancer specific death in the three cluster groups and C) Risk for disease progression for patients in the three cluster groups. [file MOL2-12-1838-s005.pdf]
