# Supplementary material for: Intratumor heterogeneity defines treatment‐resistant HER2+ breast tumors
Source: Mol Oncol. 2018 Sep 21;12(11):1838–55. doi: 10.1002/1878-0261.12375 (PMC6210052; doi:10.1002/1878-0261.12375)
Supplement: Supplementary file 6 — Fig. S6. (A) HER2 spatial organization in metastatic and non‐metastatic samples and (B) HER2 spatial organization in ER positive and ER negative samples. [file MOL2-12-1838-s006.pdf]

Supplementary Figure 6

A) HER2 spatial organization in metastatic and non-metastatic samples

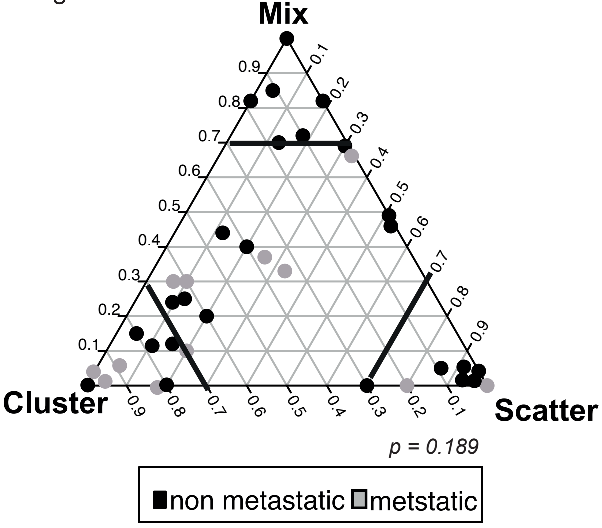

Each dot represent one sample, the position is determined by the fraction of cells with each of the three different spatial organization types (cluster, scatter or mixed, pre-treatment samples). Samples from patients later diagnosed with metastases are colored grey and those with no metastases are colored black. The number on the axis refer to the fraction of cells.

B) HER2 spatial organization in ER positive and ER negative samples

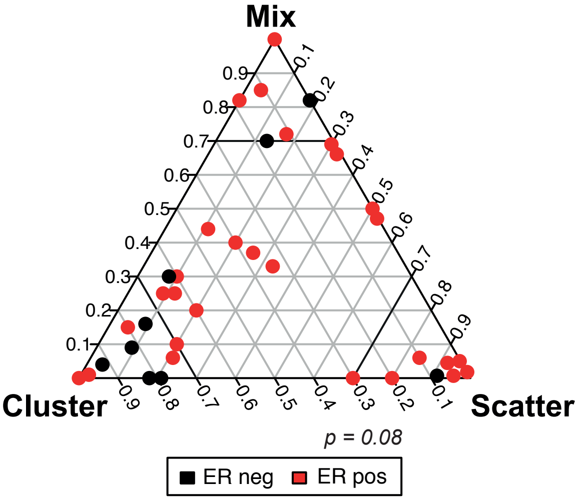

Each dot represent one sample, the position is determined by the fraction of cells with each of the three different spatial organization types (cluster, scatter or mixed, pre-treatment samples). Samples from patients wit ER+ tumors are colored red, from ER- tumors are colored black. The number on the axis refer to the fraction of cells.
