# Supplementary material for: Intratumor heterogeneity defines treatment‐resistant HER2+ breast tumors
Source: Mol Oncol. 2018 Sep 21;12(11):1838–55. doi: 10.1002/1878-0261.12375 (PMC6210052; doi:10.1002/1878-0261.12375)

Supplemental Figure 7
Survival analyses of patients with regard to A) change in phenotypic heterogeneity and B) phenotypic and genomic heterogeneity
and C) Comparison of marker assessment before and after therapy

A) Samples from patients (n=20) where the change in phenotypic groups during therapy were calculated using the KL-index. The samples were divided into equally sized groups by K-L index median and overall survival was analyzed.

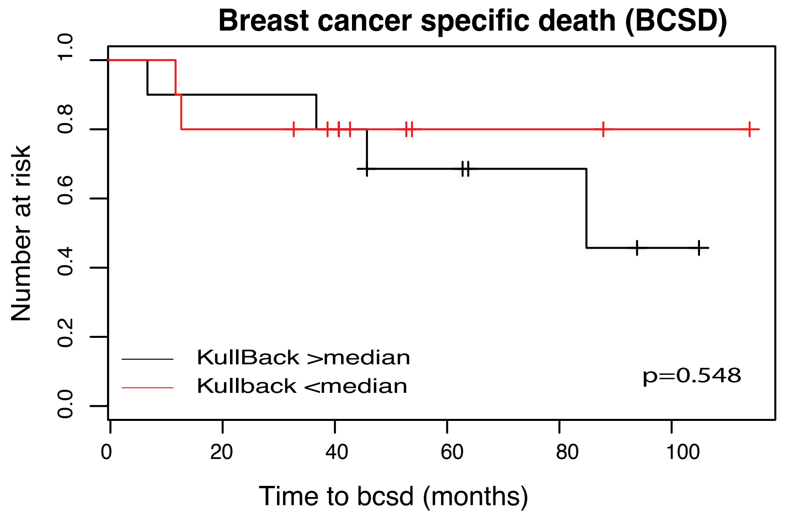

B) Samples from patients (n=20) where the change in the combined phenotypic and genomic groups during therapy were calculated using the K-L index. The samples were divided into equally sized groups by KL-index median and overall survival was analyzed.

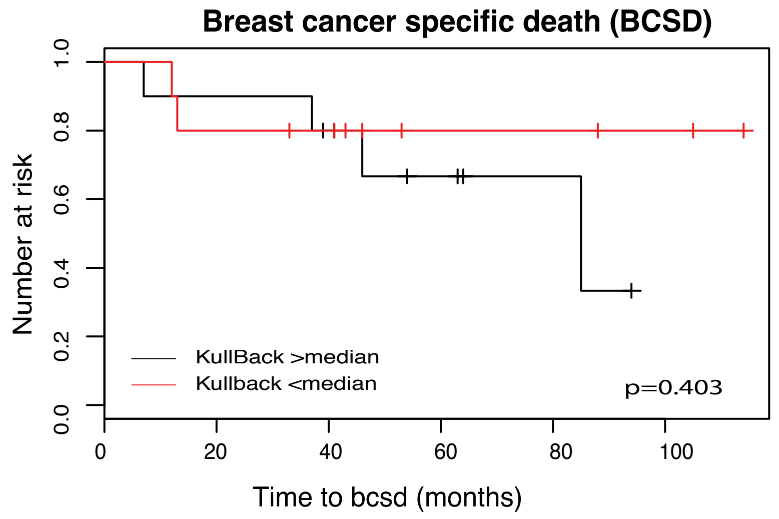

C) Samples from patients (n = 22) with non-pCR; comparison of marker assessments before and after therapy.
The y-axis represent intensity for te protein markers and area for the FISH markers.
(Pre = pre treatment biopsies, Post = post treatment biopsies).

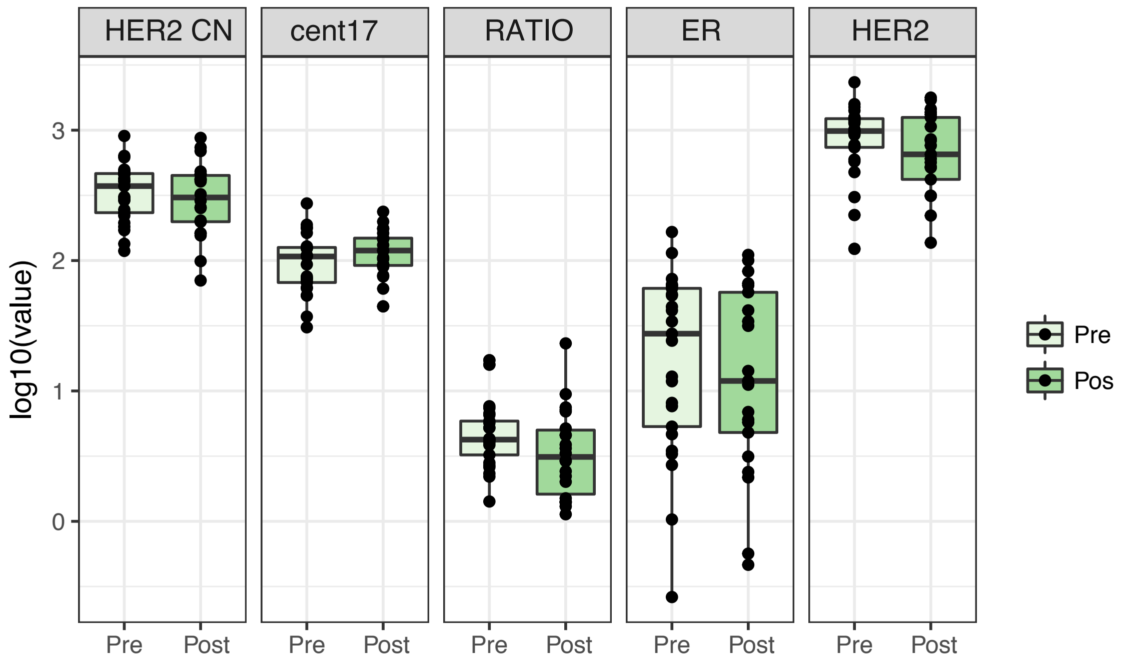

Supplement: Supplementary file 7 — Fig. S7. Survival analyses of patients with regard to (A) change in phenotypic heterogeneity and (B) phenotypic and genomic heterogeneity. (C) Comparison of marker assessment before and after therapy. [file MOL2-12-1838-s007.pdf]
