# Supplementary material for: Intratumor heterogeneity defines treatment‐resistant HER2+ breast tumors
Source: Mol Oncol. 2018 Sep 21;12(11):1838–55. doi: 10.1002/1878-0261.12375 (PMC6210052; doi:10.1002/1878-0261.12375)
Supplement: Supplementary file 8 — Table S1. Clinical and pathology data for all patients. [file MOL2-12-1838-s008.pdf]

Supplemental Table 1: Clinical and pathology data for all patients

Patient ID	PgR (IHC)	PgR (IHC)%	ER (IHC)	HER2+IHC	Histology	Grade	M	N	T	Stage	Size(mm)	ER (IFISH) %	ER (IFISH)	Lymph nodes examined	Number of metastatic lymph nodes	neoadjuvant treatment (months)	neoadjuvant response	Developed distant metastasis	Status at the time of last follow up
13	pos	1-10%	pos	3+	IDC	2	0	1	3	3	80	61,7	pos	11	5	6	SD	yes	alive
40	pos	10-50%	pos	3+	IDC	2	0	1	3	3	70	0	neg	10	0	6	PR1	no	alive
48	neg	0	neg	2+	IDC	2	0	0	3	3	80	31	pos	11	4	7	PR1	no	alive
53	pos	10-50%	pos	3+	IDC	3	0	0	2	2	65	75	pos	13	0	7	CR	no	alive
69	neg	0	neg	3+	IDC	3	0	0	3	2	110	5,2	pos	20	0	8	PR2	no	alive
6178	neg	0	pos	3+	IDC	2	0	0	4	3	22	71,6	pos	8	0	6	PR1	no	alive
6361	neg	0	neg	3+	IDC	2	0	1	3	3	65	2,3	pos	14	0	7	CR	no	alive
6370	neg	0	pos	3+	IDC	3	0	2	NA	3	30	19,4	pos	9	5	5	PR1	yes	dead
6410	neg	0	pos	3+	ILC	2	0	3	3	3	100	0	neg	11	8	6	PR1	yes	alive
6450	neg	0	neg	3+	IDC	2	0	0	3	2	100	100	Pos	3	0	6	CR	no	alive
6739	pos	10-50%	pos	3+	IDC	2	0	2	2	3	37	79,9	Pos	16	13	7	PR1	no	alive
6748	neg	0	neg	2+	IDC	3	NA	3	4	3	85	4	Pos	7	5	5	SD	yes	dead
6930	pos	10-50%	pos	3+	IDC	3	0	2	4	3	100	14,7	Pos	4	0	9	CR	yes	dead
7126	pos	10-50%	neg	3+	IDC+DCIS	3	0	1	2	2	70	8,4	Pos	6	0	7	PR1	no	alive
7334	neg	0	neg	3+	IDC	3	0	1	3	3	75	0	Neg	15	0	7	CR	no	alive
7347	pos	10-50%	pos	3+	IDC+DCIS	2	0	0	2	2	70	57,5	pos	9	0	8	CR	no	alive
7350	pos	50-100%	pos	3+	IDC	2	0	0	3	2	70	52,2	pos	11	1	5	PR1	no	alive
7360	neg	0	neg	3+	IDC	3	0	1	4	3	90	0	neg	7	0	6	PR2	yes	dead
7362	neg	10-50%	neg	2+	IDC+DCIS	2	0	0	3	2	60	49,6	pos	11	0	6	PR1	no	alive
7363	pos	10-50%	pos	3+	IDC+DCIS	3	0	1	3	3	80	93	pos	10	0	7	CR	no	alive
7364	neg	0	pos	3+	DCIS	2	0	1	0	2	NM	1,8	pos	13	0	10	CR	no	alive
7370	pos	50-100%	pos	3+	IDC	2	0	0	4	3	60	45	pos	14	1	6	PR1	no	alive
7374	neg	0	neg	3+	IDC+DCIS	3	0	1	3	3	80	0	neg	12	2	5	PR1	yes	alive
7379	pos	10-50%	pos	3+	IDC	2	0	0	3	2	80	2,7	pos	7	1	7	PR1	yes	dead
7406	pos	10-50%	pos	3+	IDC	3	0	1	4	3	100	29,3	pos	7	0	5	PR1	yes	alive
7417	neg	0	neg	3+	IDC	2	0	1	4	3	100	0	neg	16	0	7	CR	no	alive
7424	neg	0	pos	3+	IDC	3	0	1	3	3	90	17,3	pos	9	9	3	PR1	no	alive
7428	pos	1-10%	pos	3+	IDC	2	1	1	4	4	85	3,6	pos	8	1	5	PR1	yes	dead
7435	neg	0	neg	3+	IDC	3	0	1	4	3	130	1,1	pos	8	5	5	SD	yes	dead
7441	pos	50-100%	neg	2+	IDC	3	0	1	3	3	55	25,9	pos	18	0	6	PR1	no	alive
7457	neg	0	neg	3+	IDC	2	NA	1	4	NA	70	0,5	neg	16	0	6	CR	no	alive
7556	neg	0	neg	3+	IDC	2	0	2	3	3	55	0,7	neg	4	0	6	CR	no	alive
7560	neg	0	neg	2+	IDC	2	0	1	2	2	50	1,4	pos	6	0	6	PR1	no	alive
7563	neg	0	pos	3+	IDC	2	0	1	1	2	30	42,2	pos	6	0	8	PR1	yes	dead
7588	pos	50-100%	pos	3+	IDC	2	0	1	3	3	60	55,5	pos	17	0	6	PR1	no	alive
7619	neg	0	pos	2+	IDC	2	0	1	3	3	60	44,9	Pos	12	6	6	PR1	no	alive
7641	neg	0	neg	3+	IDC	2	0	0	3	2	80	0,3	Neg	10	0	7	CR	no	alive
