# Supplementary material for: Intratumor heterogeneity defines treatment‐resistant HER2+ breast tumors
Source: Mol Oncol. 2018 Sep 21;12(11):1838–55. doi: 10.1002/1878-0261.12375 (PMC6210052; doi:10.1002/1878-0261.12375)
Supplement: Supplementary file 9 — Table S2. Overview of biopsies, images and number of cells included in the study. [file MOL2-12-1838-s009.pdf]

Supplemental Table 2: Overview of biopsies, images and number of cells included in the study

	Prior treatment			Post Treatment			Metastasis		
Patient ID	Number of biopsies	Number of areas	Number of tumor cells	Number of biopsies	Number of areas	Number of tumor cells	Number of biopsies	Number of areas	Number of tumor cells
13	1	1	188	2	2	507			
40	1	1	154	1	1	64			
48	1	1	58	1	1	11			
53	1	1	317						
69	1	1	154	1	1	97			
6178	1	1	67						
6361	2	4	858						
6370	1	1	139	1	2	180			
6410	1	2	98	2	3	26			
6450	1	1	463	1	1	30			
6739	2	3	134						
6748	1	2	693	1	1	244			
6930	1	1	313				1	1	436
7126	1	1	328						
7334	1	1	28						
7347	1	1	193						
7350	1	2	90	1	3	116			
7360	1	1	67	2	2	194	1	1	154
7362	1	1	355	1	4	91			
7363	1	1	385	1	1	24			
7364	1	1	218						
7370	2	2	195	1	1	100			
7374	1	1	87	1	3	74			
7379	1	2	221	1	1	174			
7406	2	3	92	2	5	77			
7417	1	1	14						
7424	2	6	202	1	2	106			
7428	1	1	139	1	2	78			
7435	1	2	1043	1	2	626	1	2	142
7441	1	1	467	1	1	40			
7457	1	1	221						
7556	1	1	145						
7560	1	4	144	1	1	22			
7563	1	1	154	1	4	158			
7588	1	1	155	1	1	232			
7619	1	1	246	1	1	174			
7641	2	3	576						
