# Supplementary material for: Intratumor heterogeneity defines treatment‐resistant HER2+ breast tumors
Source: Mol Oncol. 2018 Sep 21;12(11):1838–55. doi: 10.1002/1878-0261.12375 (PMC6210052; doi:10.1002/1878-0261.12375)
Supplement: Supplementary file 10 — Table S3. Clinico‐pathological demographics for the different cluster groups. [file MOL2-12-1838-s010.pdf]

Supplemental Table 3: Clinico-pathological demographics for the different cluster groups

	Phenotypic ER/HER2 n=37			Genomic *HER2* cn n=37			Phenotype+ Genotype ER/HER2 + HER2cn n=37		
	Cluster P1	Cluster P2	Cluster P3	Cluster 1	Cluster 2	Cluster 3	Category 1	Category 2	Category 3
ER pos	11	14	3	6	11	11	8	8	12
ER neg	0	9	0	1	3	5	1	0	8
	Fisher test:. p=0.03								
ER neg	0	9	0	1	3	5	1	0	8
ER 1-10%	0	9	0	0	5	4	7	0	3
ER 10-50%	2	5	3	4	1	5	0	0	9
ER >50%	9	0	0	2	5	2	1	8	0
	Fisher.test:p<0.00005						Fisher test: p<0.000002		
PR neg	4	17	1	2	10	10	4	2	16
PR pos	7	6	2	5	4	6	5	6	4
	Fisher test:p=0.059						Fisher test: p=0.016		
HER2 IHC 2+	1	3	2	3	1	2	4	0	2
HER2 IHC 3+	10	20	1	4	13	14	5	8	18
	Fisher test. p=0.077						Fisher test: p=0.03		
Grade2	10	11	2	5	7	11	7	6	10
Grade3	1	12	1	2	7	5	2	2	10
	Fisher.test: p=0.036								
Stage 2	5	7	0	1	6	0	2	4	6
Stage 3	6	14	3	4	9	0	7	4	12
Stage 4	0	1	0	7	3	1	0	0	1
									n=36
IDC	8	19	3	7	14	14	8	6	16
IDC+DCIS	2	3	0	0	0	1	1	2	3
ILC	0	1	0	0	0	1	0	0	1
pCR	2	10	0	1	7	4	0	4	8
non pCR	9	13	3	6	7	12	9	4	12
							Fisher test: p=0.035		
Alive	10	16	3	7	12	10	8	8	13
dead	1	7	0	0	2	6	1	0	7
							Fisher test: p=0.1158		
not met	8	14	3	6	12	7	6	8	11
Met	3	9	0	1	2	9	3	0	9
	Fisher test:p=0.03			Fisher test: p=0.029			Fisher test: p=0.077		
CR	5	6	1	1	7	4	0	4	8
PR1	5	13	2	5	4	11	8	4	8
PR2	0	2	0	0	1	1	0	0	2
SD	1	2	0	1	2	0	1	0	2
	n=11	n=23	n=3	n=7	n=14	n=16	n=9	n=8	n=20
