# Supplementary material for: Intratumor heterogeneity defines treatment‐resistant HER2+ breast tumors
Source: Mol Oncol. 2018 Sep 21;12(11):1838–55. doi: 10.1002/1878-0261.12375 (PMC6210052; doi:10.1002/1878-0261.12375)
Supplement: Supplementary file 11 — Table S4. Shannon index calculated for each case. [file MOL2-12-1838-s011.pdf]

Supplemental Table 4: Shannon index calculated for each case. Below are the mean values per P, G and PG groups

| ID | clustPhenO | clustGen | clustPG | GenEnt | PhenEnt | GenPhenEnt |
|---|---|---|---|---|---|---|
| 13 | P1 | G1 | PG1 | 0,88486105 | 1,067364192 | 1,937747451 |
| 40 | P2 | G1 | PG1 | 1,072145215 | 0,511016782 | 1,579188049 |
| 48 | P2 | G1 | PG1 | 0,973555695 | 0,727634526 | 1,674284633 |
| 7370 | P3 | G1 | PG1 | 1,084362951 | 0,976027345 | 1,757741189 |
| 7441 | P3 | G1 | PG1 | 1,044271213 | 0,572114114 | 1,61633061 |
| 7619 | P3 | G1 | PG1 | 1,061358383 | 1,193088504 | 2,224236362 |
| 7362 | P1 | G3 | PG1 | 1,005082351 | 0,724033338 | 1,699914074 |
| 7563 | P1 | G3 | PG1 | 1,069365985 | 0,857193871 | 1,895418822 |
| 7406 | P2 | G3 | PG1 | 1,035455319 | 0,842941776 | 1,848582709 |
| 53 | P1 | G1 | PG2 | 1,020840375 | 0,754438107 | 1,713146691 |
| 6178 | P1 | G2 | PG2 | 0,586610888 | 0,654777095 | 1,174763805 |
| 6450 | P1 | G2 | PG2 | 0,748229515 | 0,256922758 | 0,954160867 |
| 6739 | P1 | G2 | PG2 | 0,58698734 | 0,502464571 | 1,06824907 |
| 7347 | P1 | G2 | PG2 | 0,586041443 | 0,748492937 | 1,274449944 |
| 7363 | P1 | G2 | PG2 | 0,702232759 | 0,266271834 | 0,945602124 |
| 7350 | P1 | G3 | PG2 | 0,936295817 | 0,744931212 | 1,627519549 |
| 7588 | P1 | G3 | PG2 | 0,835007014 | 0,720841946 | 1,482775942 |
| 69 | P2 | G2 | PG3 | 0,407557109 | 0,272829603 | 0,666436607 |
| 6361 | P2 | G2 | PG3 | 0,707587332 | 0,110655599 | 0,817166899 |
| 6748 | P2 | G2 | PG3 | 0,697406792 | 0,188865729 | 0,88113603 |
| 7126 | P2 | G2 | PG3 | 0,603776201 | 0,315327938 | 0,900122384 |
| 7334 | P2 | G2 | PG3 | 0,469220348 | 0,257318641 | 0,711902542 |
| 7424 | P2 | G2 | PG3 | 0,688192474 | 0,844762215 | 1,482404499 |
| 7435 | P2 | G2 | PG3 | 0,730194576 | 0,156843334 | 0,879376121 |
| 7457 | P2 | G2 | PG3 | 0,48038335 | 0,080487507 | 0,539788108 |
| 7556 | P2 | G2 | PG3 | 0,509873135 | 0,041195017 | 0,549985124 |
| 6370 | P2 | G3 | PG3 | 0,838199801 | 0,49231948 | 1,285579224 |
| 6410 | P2 | G3 | PG3 | 0,967706007 | 0 | 0,967706007 |
| 6930 | P2 | G3 | PG3 | 0,955499949 | 0,417407996 | 1,370736633 |
| 7360 | P2 | G3 | PG3 | 0,724227326 | 0 | 0,724227326 |
| 7364 | P2 | G3 | PG3 | 0,757204614 | 0,091540729 | 0,8428913 |
| 7374 | P2 | G3 | PG3 | 0,806452735 | 0,109452358 | 0,89559227 |
| 7379 | P2 | G3 | PG3 | 0,934292781 | 0,458120484 | 1,340846865 |
| 7417 | P2 | G3 | PG3 | 1,034601233 | 0,519579839 | 1,195268417 |
| 7428 | P2 | G3 | PG3 | 0,885850033 | 0,452764185 | 1,253586758 |
| 7560 | P2 | G3 | PG3 | 0,793732915 | 0,341938357 | 1,091248356 |
| 7641 | P2 | G3 | PG3 | 0,959751389 | 0,568747866 | 1,518832647 |

| clustergroup | mean SI |
|---|---|
| G1 | 1,02 |
| G2 | 0,6 |
| G3 | 0,9 |
| P1 | 0,66 |
| P2 | 0,34 |
| P3 | 0,91 |
| PG1 | 1,8 |
| PG2 | 1,28 |
| PG3 | 0,99 |
