# Supplementary material for: Intratumor heterogeneity defines treatment‐resistant HER2+ breast tumors
Source: Mol Oncol. 2018 Sep 21;12(11):1838–55. doi: 10.1002/1878-0261.12375 (PMC6210052; doi:10.1002/1878-0261.12375)
Supplement: Supplementary file 12 — Table S5. Relationship between the different HER2 spatial patterns and (A) response to therapy, (B) metastasis, (C) ER status and (D) ER percentage. [file MOL2-12-1838-s012.pdf]

Supplemental Table 5: Repationship between HER2 spatial patterns and A) response to therapy, B) metastasis, C) ER status and D) ER percentage.

A) Pre-treatment Samples (n=37) and response to therapy

	pCR	non pCR
Cluster	4	6
Mix	5	1
Scatter	0	8
<70%	3	10

Fisher test: p=0.007

B) Pre-treatment Samples (n=37) and disease progression

	met	non Met
Cluster	5	5
Mix	0	6
Scatter	2	6
<70%	5	8

Fisher test: p=0.189

C)

Pre-treatment samples (n=37) and ER status

	ER pos	ER neg
Cluster	5	5
Mix	4	2
Scatter	7	1
<70%	12	1

Fisher test: p=0.08

D) Pre-treatment samples (n=37) grouped by ER percentage

	neg	1-10%	10-50%	>50%
Cluster	5	5	0	0
Mix	2	1	1	2
Scatter	1	0	5	2
<70%	1	3	4	5

Fisher test: p=0.007
