# Supplementary material for: Intratumor heterogeneity defines treatment‐resistant HER2+ breast tumors
Source: Mol Oncol. 2018 Sep 21;12(11):1838–55. doi: 10.1002/1878-0261.12375 (PMC6210052; doi:10.1002/1878-0261.12375)
Supplement: Supplementary file 13 — Table S6. Clinico‐pathological demographics for Kullback Leibler groups. [file MOL2-12-1838-s013.pdf]

Supplemental Table 6: clinico-pathological demographics for Kullback Leibler groups

	low KL	high KL
ER.pos	2	2
ER.neg	8	8
	Fisher.test: P=1	
ER neg	2	2
ER 1-10%	4	1
ER 10-50%	3	5
ER >50%	1	2
	Fisher.test: P=0.5	
PR.neg	5	7
PR.pos	5	3
	Fisher.test: p=0.64	
HER2.IHC 2+	2	3
HER2.IHC 3+	8	7
	Fisher.test: p=1	
Grade2	4	7
Grade3	6	3
	Fisher.test : p=0.36	
stage.2	4	1
stage.3	6	9
	Fisher.test: p=0.3	
IDC	10	9
ILC	0	1
	Fisher.test: p=1	
LK.post.neg	6	2
LK.post.pos	4	8
	Fisher.test: p=0.16	
	n=10	n=10

	low KL	high KL
Alive	5	9
dead	5	1
	Fisher.test: p=0.14	
not met	4	6
Met	6	4
	Fisher.test: p=0.65	
PR1	6	9
PR2	2	0
SD	2	1
	Fisher.test: p=0.37	
GenClust1	2	4
GenClust2	3	1
GenClust3	5	5
	Fisher.test: p=0.44	
PhenClust1	2	2
PhenClust2	7	6
PhenClust3	2	2
	Fisher.test: p=1	
PhenGenClust1	4	5
PhenGenClust2	5	4
PhenGenClust3	1	1
	Fisher.test: p=1	
	n=10	n=10
